# Integrating *N*-glycan and CODEX imaging reveal cell-specific protein glycosylation in healthy human lung†

**DOI:** 10.1039/d4mo00230j

**Published:** 2025-05-20

**Authors:** Dušan Veličković, Jeffrey Purkerson, Harsh Bhotika, Heidie Huyck, Geremy Clair, Gloria S. Pryhuber, Christopher Anderton

**Affiliations:** a Earth and Biological Sciences Directorate, Pacific Northwest National Laboratory Richland Washington USA dusan.velickovic@pnnl.gov; b Department of Pediatrics, University of Rochester Medical Center Rochester New York USA

## Abstract

Identifying cell-specific glycan structures in human lungs is critical for understanding the chemistry and mechanisms that guide cell–cell and cell–matrix interactions and determining nuanced functions of specific glycosylation. Our dual-modality omics platform, which uses matrix-assisted laser desorption/ionization (MALDI) mass spectrometry imaging (MSI) to profile glycan chemistry at 50 μm × 50 μm scale, combined with co-detection by indexing (CODEX) to provide cell identification from the exact same tissue section, is a significant step in this direction. It enabled us to detect, differentiate, and reveal chemical properties of *N*-glycans in the various cell types of a human lung, suggesting the cell-specific function of distinct carbohydrate moieties. This innovative technological combination bridges the gap between the specific protein glycosylation and their cellular origin, paving the way for targeted studies in the lungs and many other human tissues where glycans mediate cell–cell recognition events.

## Abstract 2

Identifying cell-specific glycan structures in human lungs is critical for understanding the chemistry and mechanisms that guide cell–cell and cell–matrix interactions and determining nuanced functions of specific glycosylation.

## Graphical abstract

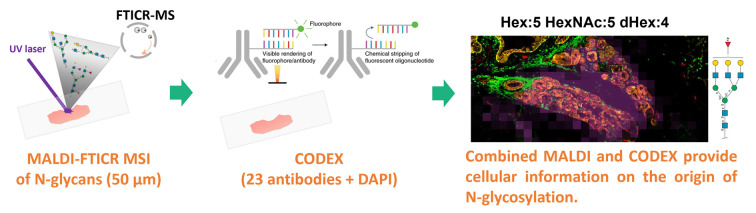

## Introduction

Protein *N*-glycosylation is a ubiquitous post-translational modification that plays essential biological roles in vital molecular processes ranging from protein quality control, protein clearance, and intracellular trafficking to various cell–cell recognition events that include cell adhesion, receptor activation, self/non-self-recognition, and host–pathogen interaction.^1,2^ However, much of our knowledge of human glycans is confined to those found on blood cells, free in plasma, and attached to antibodies.^3^ We still know very little about cell-specific glycosylation differences in human tissues, including within the lung.

Current evidence suggests human lungs have a very complex glycome, with over 500 *N*-glycan structures regulating tissue development and interactions with inhaled pathogens.^3^ Based on their structure, *N*-glycans are classified as pauci-mannose, high-mannose, hybrid, and complex *N*-glycans, which can be additionally decorated with various specialized monosaccharides (*i.e.*, fucose, galactose, and sialic acid) and have distinct branching patterns that will impact their function and interaction with the environment.^4^ More specifically, complex *N*-glycans can have bisecting and tetra-antennary organization, polylactosamine and sialic acid decorations, core and antenna fucosylation, or combinations of these.^5^ However, the glycan structures involved in cell and tissue physiological and pathological processes remain elusive. This elusiveness is partly due to insufficient information on their localization, cell type specificity, and protein carriers.

Matrix-assisted laser desorption/ionization mass spectrometry imaging (MALDI-MSI) has become a prime technique for revealing protein *N*-glycan composition and localization within biological tissues.^6–8^ In this approach, *N*-glycans are enzymatically released from their glycoproteins in an *in situ* fashion, after which a standard MALDI workflow can be followed, where a UV laser is used to ablate and ionize *N*-glycans from the tissues in a spatially defined pattern. Ionized *N*-glycans are then introduced into a mass spectrometer to measure their molecular weight, which inform on their chemical formula and putative identification. This results in the ability to measure and map the relative abundance of these *N*-glycans across tissues in an untargeted, highly multiplexed fashion. Typical applications achieve lateral resolution of tens of microns or less, where approaching cellular resolution in human tissues is possible.^7^ Compared to lectin and other glycan-binding tissue staining protocols, which can only partially characterize glycan composition (*i.e.*, specific epitope), MALDI-MSI is broad, untargeted, measures an exact mass of the *N*-glycans, and, in combination with specialized databases,^9^ reveals *N*-glycans’ composition (*e.g.*, type and number of monosaccharides) in a particular area of the tissue, which is ideal for biomarker discovery and mechanistic studies.^10^ For example, we recently discovered that specific *N*-glycans are glomerulosclerosis biomarkers in diabetic kidney disease (DKD) kidneys using MALDI-MSI.^7^

Compatibility of MALDI-MSI-based spatial *N*-glycomics with other imaging modalities affords going beyond just the creation of distributional maps of *N*-glycans to put findings into biological, physiological, or pathological contexts. The most readily applied approach is performing histochemical (HC) and immunohistochemical (IHC) staining after performing spatial *N*-glycomics, which, for example, revealed *N*-glycan signatures of immune cell populations in lung tissue after COVID-19 infection^11^ and alterations in pulmonary *N*-glycans following irradiation.^12^

Tissues, such as those in the lung, have complex architectures and cellular organization, and HC and IHC are often insufficient in describing the entire cell repertoire. Because those tissues are composed of various cell types in close association, multiplexed antibody imaging technologies have become highly desired for single-cell level characterization.^13^ One emerging technique that provides a deep view into the single-cell spatial relationships and disease progression is co-detection by indexing (CODEX),^14,15^ which relies on DNA-conjugated antibodies and the cyclic addition and removal of complementary fluorescently labeled DNA reporters. Compared to traditional IHC, which typically assesses only a limited number of protein markers (two to four) in a tissue section, CODEX can visualize 30 or more markers *in situ*, refining spatial cell type identification.^14^

Herein, we took advantage of the cellular resolution the previously designed CODEX v.2 immunohistochemical imaging panel provides,^16–18^ and combined it with MALDI-MSI-based spatial *N*-glycomics on the same human lung tissue section. In this innovative technological combination, MALDI-MSI generated *N*-glycome profiles of tissue regions comprising many structures and cell types, and CODEX helped us determine the potential cellular origin of these protein post-translational modifications. Moreover, using our newly developed algorithm for *N*-glycan classification based on their composition, we revealed the chemical properties of *N*-glycans enriched in the specific cell types of a human lung, generating hypotheses for the potential functional role of the specific carbohydrate moieties.

## Materials and methods

### Lung tissue source

Transplant-quality donor lung that could not be matched to an organ recipient was obtained through the United Network of Organ Sharing *via* the National Disease Research Interchange (NDRI) and the International Institute for Advancement of Medicine (IIAM). The donor was a 23-year-old white male with no past medical history, and the cause of death was brain injury. The organ recovery, storage during transport and processing into the BioRepository for INvestigation of the Lung (BRINDL, https://brindl.urmc.rochester.edu) were performed as described previously^19^ and available at protocol.io.^20^ Lung sample was reviewed by pathologists for quality assessment before enrolling in this study (ESI,† Fig. S1), showing normal lung structure, patchy mild fibrin and periairway lymphocytic inflammation and macrophage accumulation, with warm and cold ischemic times of 0 h and 31 h, respectively. The University of Rochester IRB approved and oversees this study (IRB approval# RSRB00047606).

### Mass spectrometry imaging of *N*-glycans

Step-by-step details of the method can be found in protocols.io.^21^ Briefly, a FFPE block of human lung tissue were prepared using the protocol,^22^ sectioned at 5 μm thickness and mounted on indium tin oxide (ITO)-coated glass slides. Slides were heated, dewaxed by xylene washes, and rehydrated in serial ethanol (EtOH)/water (v/v) washings and then subjected to antigen retrieval in boiling citraconic buffer followed by PNGase F (N-Zyme Scientifics, 100 μg mL^−1^) spraying using a M5 Sprayer (HTX Technologies), and sample incubation in a relative humidity of 89% for 2 h at 37 °C, as described previously.^23^

After incubation, α-cyano-4-hydroxycinnamic acid (CHCA, Sigma-Aldrich) – 7 mg mL^−1^ (50% ACN and 0.1% TFA in water (v/v)) – was sprayed over the tissue sections using the M5 Sprayer, as described previously.^7^ MALDI-MSI experiments were performed using a scimaX 7 Tesla Magnetic Resonance Mass Spectrometer (MRMS; Bruker Daltonics) equipped with a dual ESI/MALDI ion source and a Smart-beam II Nd:YAG (355 nm) laser. The instrument was operated in 1 w, positive ion mode over an *m*/*z* range of 1000–5000 with an estimated resolving power of 120 000 at *m*/*z* 400. The target plate stepping distance (lateral resolution) was 50 μm. The ion *m*/*z* 1809.6393 ([M + Na]^+^ of Hex5 dHex1 HexNAc4) was used as a lock mass for on-line calibration. Imaging data were acquired using FlexImaging (v 4.1, Bruker Daltonics).

### Creating glycan mining and ontology (*N*-glycan MiniOn) to automate the dissection of the *N*-glycan composition

**Fig. 1:**
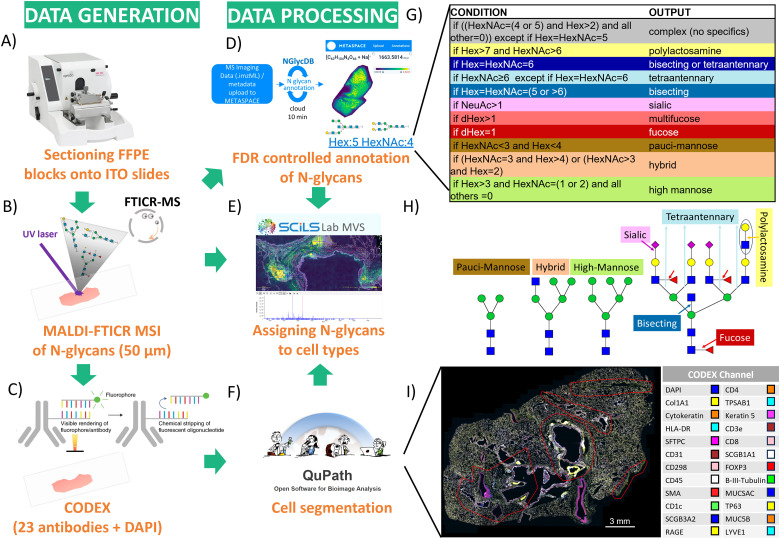
The workflow for integrating MALDI-MSI-based spatial *N*-glycomics and CODEX analysis of the same lung tissue section. (A) The FFPE block of the lung tissue was sectioned, and the same section was used for both (B) *N*-glycan MALDI-MSI and (C) CODEX assays. (D) METASPACE was used for *N*-glycan compositional annotation, and (E) SCiLS for integration with (F) CODEX images visualized and segmented using QuPath. (G) Based on their chemical similarities, a classification algorithm for converting *N*-glycan composition to *N*-glycan class was performed to group *N*-glycans. (H) Symbol nomenclature for *N*-glycans highlighting specific structural features. (I) CODEX image, with 24 channels active, generated in QuPath and exported into SCILS for overlaying with *N*-glycan MALDI MSI data. Closed curved red lines in the tissue outline regions where MALDI-MSI was performed.

To streamline the classification of the *N*-glycans in the different classes, we developed an R package named glycan mining and ontology (*N*-glycan MiniOn) to automate the dissection of the *N*-glycan composition. This open-source package is available on GitHub (https://github.com/GeremyClair/NglycanMiniOn/). This package is similar to what we previously developed to generate lipid ontologies from the lipid names (Lipid MiniOn).^24^ In *N*-glycan MiniOn, the function ‘NGlycan_miner()’ parses the names of *N*-glycans to enable their attribution to different classes. First, it identifies the nature and the exact number of monosaccharides composing the *N*-glycans. Next, using the rules described in Fig. 1(G), it identifies the classes each *N*-glycan belongs to. Once this information is parsed, a second function, ‘Nglycan_ontologies()’, generates a list of ontology terms associated with each *N*-glycan enabling to perform enrichment analyses using popular methods such as Fisher's exact test, DAVID's modified Fisher's exact test (EASE score),^25^ binomial test, or Kolmogorov–Smirnov test for ranked-based enrichment analyses. It also enables the generation of Figures depicting the frequency of *N*-glycans in different clusters. A shiny app was developed to streamline the analysis of *N*-glycan structures for non-coding researchers (https://github.com/GeremyClair/NglycanMiniOn_shiny).

### CODEX immunohistochemical imaging

Post-MALDI imaging CODEX IHC protocol is available in protocols.io.^26^ Briefly, the MALDI-matrix was removed from lung sections through 2 × 2 min incubation in 50% ACN and rehydrated *via* a decreasing ethanol series prior to high pH Heat-Induced Epitope Retrieval (HIER). After cooling, lung tissue was washed with hydration buffer (Akoya Biosciences) and then incubated for 20–30 min in staining buffer (Akoya). Sections were then covered with 200 μL of antibody buffer composed of staining buffer supplemented with recommended concentrations of N, G, J & S blockers (Akoya Biosciences) and the specified dilutions of up to 34 antibody–barcode conjugates followed by incubation in a humidified chamber at room temperature for 3 h. Sections were then washed with staining buffer followed by fixation in 1.6% paraformaldehyde for 10 min After a series of rinsing in PBS, samples were placed in a storage buffer (Akoya Biosciences) and photobleached by illumination with a 200 mA, 15 watts, 1600 lumens bulb overnight at 4 °C. Image acquisition was performed using the Phenocycler-Fusion 1.0 platform utilizing the 20× (0.5 μm per pixel) objective and the Fusion 1.0.8 software according to manufacturer recommendations, as described.^26^ Negative control staining using barcodes without conjugated antibodies was performed as QC to ensure there was no off-target barcode staining. Our validation and QC process is detailed in our antibody validation protocol.^27^

### Integration of MALDI-MSI and CODEX on the same tissue section

MALDI-MS imaging data files were imported into the SCiLS (Bruker Daltonics) software and exported to imzML. The resulting .imzML and .ibd files were submitted to METASPACE for data processing and *N*-glycan annotation, using the NGlycDB V1 as the database.^9^ METASPACE was used for data visualization, where the “Show representative spatial patterns for dataset” tool was used to select distinct spatial patterns (ESI,† Fig. S2). A *m*/*z* list of detected *N*-glycans, their annotations at a compositional level, and visualization of their spatial distribution were created using the METASPACE annotation platform, providing information on possible *N*-glycan isomeric structures for each ion image. A *m*/*z* list of annotated *N*-glycans was imported back into the SCiLS for integration with CODEX images. CODEX data were opened in QuPath bioimage analysis software and exported as rendered RGB images using available channels.^28^ Cell segmentation was performed utilizing the StarDist extension^29,30^ in QuPath. CODEX images were imported into SCiLS and overlayed with MALDI-MSI ion images created using an annotated *m*/*z* list from METASPACE.

## Results

### MALDI-MSI: one hundred fifty *N*-glycans are distributed across different areas of lung tissue

As a proof-of-concept of our technology, illustrated in Fig. 1, we used *N*-glycan MALDI-MS imaging protocol optimized in our previous work,^23^ where we established that enzyme digestion at 89% relative humidity (maintained by a saturated KNO_3_ solution) minimizes diffusion (*i.e.*, delocalization of molecules from their endogenous locations) of the released *N*-glycans from their parent glycoprotein without sacrificing the number and sensitivity of detected *N*-glycans. Herein, we detected more than 150 *N*-glycans with various spatial patterns associated with different anatomical regions in an FFPE section of the left upper lobe of a human lung.

Insight into the compositions, tentative structures, and localization of *N*-glycans can be visualized using the METASPACE platform, where *N*-glycan images are registered and overlayed on a high-resolution microscopy image: https://metaspace2020.eu/project/velickovic-2024_MALDI_CODEX. Manual inspection of data and using the “Show representative spatial pattern for datasets” tool in METASPACE immediately revealed several characteristic spatial patterns of *N*-glycan ion images that align with different anatomical regions in the lung, including adventitial regions of airways and blood vessels, submucosal glands, cartilaginous shields, the smooth muscle of pulmonary artery, and alveolar parenchyma (ESI,† Table S1). This spatial heterogeneity and diversity of protein *N*-glycans suggests that these post-translational modifications might play distinct roles in these respective lung tissue functional units.

### A new *N*-glycan classification algorithm revealed common carbohydrate epitopes in the lung tissue functional units

We then aimed to gain additional insight into the potential structure–function relationship of these protein *N*-glycans. First, using the co-localization analysis, we created a list of *N*-glycans (*i.e.*, their *m*/*z* and composition) that belong to each spatial structural cell pattern (specifically, anatomical region of the lung) (ESI,† Table S1). Second, to reveal common *N*-glycan chemical moieties enriched in distinct anatomical/functional regions, we constructed a classification rule (Fig. 1(G)) that converts the *N*-glycan composition (*e.g.*, number and type of monosaccharides, such as Hex:5 HexNAc:4, Fig. 1(D)) to the *N*-glycan structural characteristics (*e.g.*, bisecting, fucosylated, *etc.*). Thanks to the canonical *N*-glycan structural organization, *N*-glycan composition often reveals much about *N*-glycan structure despite multiple structural isomers that can be associated with the given composition. Herein, we used *N*-glycan compositions and their available structures in NGlycDB within METASPACE^9^ to generate a general classification rule that, based on the number and identity of monosaccharides, defined *N*-glycan as pauci-mannose, hybrid, high-mannose, fucosylated, multi-fucosylated, sialylated, bisecting, tetra-antennary, polylactosamine, or complex without any of these specific moieties.

Establishing rules for fucosylated, multi-fucosylated, and sialylated *N*-glycans is straightforward, as these monosaccharides are unique and present in the *N*-glycan composition as dHex:1, dHex > 1, and NeuAc ≥ 1, respectively. High mannose glycans consist exclusively of mannoses (hexoses, denoted as Hex) attached to the Hex:3 HexNAc:2 core. Therefore, for a glycan to be classified as high-mannose, the number of Hex must be greater than 3, and no other monosaccharides (except the two HexNAc from the core or, in rare cases, one HexNAc) should be present in the composition. Pauci-mannose *N*-glycans are truncated mannose glycans with a full (Hex:3 HexNAc:2) or partial core and may contain fucose (dHex). Consequently, for these glycans, the total number of Hex must be lower than 4, and the number of HexNAc must be lower than 3. Polylactosamine glycans are characterized by repeating lactosamine units (galactose-*N*-acetylglucosamine, Hex-HexNAc) attached to the core of complex *N*-glycans. Given that complex *N*-glycans have a minimum of 4 HexNAc (2 from the core and 2 on the core mannoses), the number of HexNAc in polylactosamine must be greater than 6, and consequently, the number of Hex must be greater than 7. Determining rules for branching features, such as bisecting and tetra-antennary glycans, is challenging and involves labor-intensive database cross-validation, as these features are not immediately apparent based solely on the *N*-glycan composition. A distinguishing characteristic of complex bisecting *N*-glycans is the presence of HexNAc attached to the central mannose of the core (Hex:3 HexNAc:2). An inspection and cross-validation of rules and bisecting structures in *N*-glycan databases reveal that bisecting *N*-glycans have an equal number of HexNAc and Hex residues, and that that number is either 5 or it is greater than 6. Tetra-antennary glycans, which require at least 4 HexNAc residues (for four antennas) attached to the core (Hex:3 HexNAc:2), are positively annotated by implementing the rule that the number of HexNAc is 6 (4 + 2) or greater. One ambiguity occurs with *N*-glycans that, in their composition, contain six hexoses and six *N*-acetylhexosamines. Such *N*-glycans can be ascribed to either bisecting or tetra-antennary structures, and without orthogonal studies, it is impossible to assign correct structural organization. In many cases, *N*-glycan composition implies that its structure contains multiple decorations, where, for example, *N*-glycan Hex:8 HexNAc:8 dHex:2 is classified as a complex bisecting-multifucose-polylactosamine-tetra antennary *N*-glycan. Our algorithm is fully available for review, and it could be modified in the future to add supplementary classes of glycans based on additional definitions.

**Fig. 2:**
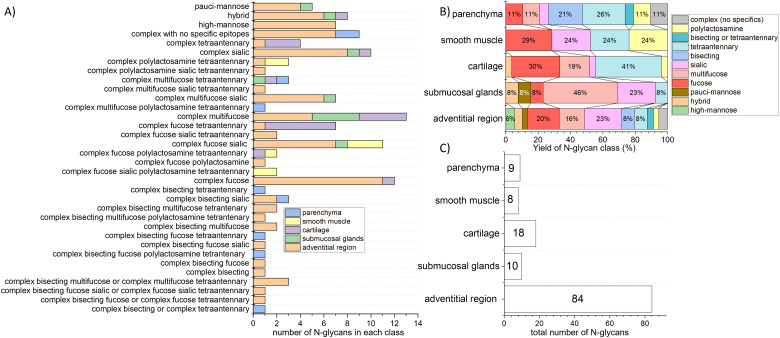
The diversity and abundance of *N*-glycan structures in human lung tissue regions were revealed through MALDI-MSI analysis and the *N*-glycan classification rule. (A) The number of *N*-glycans in each *N*-glycan class detected in each lung section anatomical region. (B) The percentile of each *N*-glycan class in each lung section anatomical region. This was calculated per the total number of *N*-glycans detected in the respective anatomical region. (C) The total number of *N*-glycans in the lung anatomical regions.

Using our classification incorporated in *N*-glycan MiniOn R package, we observed that glycoproteins with high mannose glycans are exclusively present in the adventitial region of airways and blood vessels, and glycoproteins with complex fucosylated and multifucosylated *N*-glycans are the most numerous structures in the lung. Moreover, some *N*-glycan classes are widespread and colocalize with multiple anatomical regions, including (i) hybrid, complex sialic, and complex multi-fucosylated *N*-glycans that colocalize with the airways and blood vessels adventitial regions, submucosal glands, and cartilage, (ii) complex muti-fucose-tetra antennary structures that colocalize with submucosal glands, cartilage and parenchyma, and (iii) complex fucose-sialic structures that colocalize with airways and blood vessels adventitial regions, submucosal glands, and smooth muscle layers (Fig. 2(A)). Other classes of *N*-glycans are particular for specific regions in the lung. For example, the smooth muscle layer is abundant in glycoproteins with complex fucose-sialic-polylactosamine-tetra-antennary *N*-glycan decorations. At the same time, protein *N*-glycans overrepresented in parenchyma include bisecting tetra-antennary structures with and without fucose and/or polylactosamine decorations. Notably, some of these classes are composed of only one glycan structure (Fig. 2(A)).

Upon investigation of the protein *N*-glycan profile of individual regions (Fig. 2(B)), our data shows that the adventitial region of airways and blood vessels contain the highest diversity of the *N*-glycans, with fucosylated, multi-fucosylated, and sialic acid *N*-glycans representing two-thirds of all such structures. This high diversity of *N*-glycans within the adventitial region is unsurprising, given that we detected the highest number of *N*-glycan structures colocalizing with this anatomical region (Fig. 2(C)). On the contrary, smooth muscle layers have only eight highly specialized *N*-glycans that contain fucose, sialic acid, tetraantennary, and polylactosamine structures (or their combinations; Fig. 2(C)). Finally, multi-fucosylated *N*-glycans are enriched in the submucosal glands, while cartilage dominates in tetra-antennary structures (Fig. 2(B)).

### Codex identified the cellular origin of *N*-glycans

**Fig. 3:**
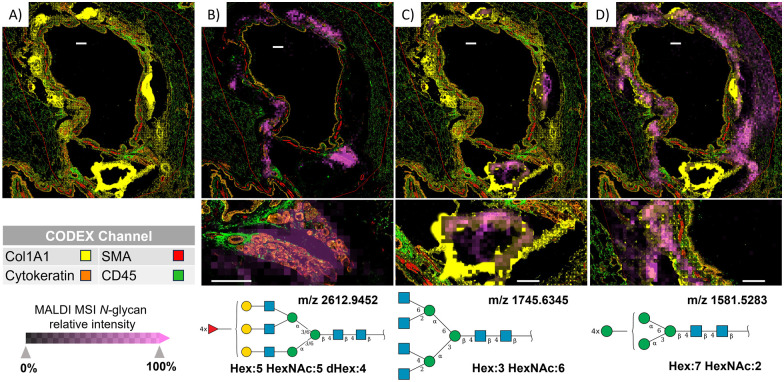
Combining MALDI-MSI and CODEX revealed *N*-glycans and the cell types they are expressed in around the bronchus. (A) The CODEX image and CODEX channel color legend (B) MALDI-MS image (magenta pixels) of Hex:5 HexNAc:5 dHex:4 (*m*/*z* 2612.9452) overlaid on CODEX image shows the high abundance of this *N*-glycan in submucosal glands, where pan-cytokeratin rather than CD45 is highly expressed. SNFG structure of this complex multifucose *N*-glycan is shown. (C) Overlayed MALDI-MS image of Hex:3 HexNAc:6 (*m*/*z* 1745.6345) over CODEX images shows the highest abundance of this *N*-glycan in cartilage, where Col1A1 is highly abundant. SNFG structure of this complex tetra antennary *N*-glycan is shown. (D) Overlayed MALDI-MS image of Hex:7 HexNAc:2 (*m*/*z* 1581.5283) over CODEX images shows the high abundance of this *N*-glycan in multiple cell types around the bronchus. SNFG structure of this high-mannose *N*-glycan is shown. White scale bars represent 300 μm.

Overlaying MALDI-MS ion images of *N*-glycan distributions with CODEX images acquired from the same section allowed us to decipher the cellular origin of these post-translational modifications in more detail. This was especially true for *N*-glycans surrounding airways, where multiple cell types occur (Fig. 3). The CODEX image in Fig. 3(A) shows protein markers around the bronchus, while the magenta pixels in Fig. 3(B)–(D) show the localization of selected *N*-glycans in the same region. We can observe that multi-fucosylated *N*-glycan (*m*/*z* 2612.9452) originates from epithelial cells rather than immune cells in submucosal glands (high co-localization of *N*-glycan signal with pan-cytokeratin marker compared to low co-localization with CD45 marker, Fig. 3(B)), while tetra antennary *N*-glycan (*m*/*z* 1745.6345) originates from cartilage and perichondral fibroblasts where Col1A1 marker is highly expressed (Fig. 3(C)). Note that the very bright Col1A1 islands are the result of the MALDI imaging workflow that led to partial detaching, folding, and condensation of cartilaginous plaques (ESI,† Fig. S3), where we can also visualize ablation marks from the MALDI laser. Further, high-mannose *N*-glycans (*m*/*z* 1581.5283) surrounding the bronchus (Fig. 3(D)) are associated with collagenous structures rather than the adjacent epithelium and smooth muscle cells, as those *N*-glycans were not observed in the vicinity of cells expressing pan-cytokeratin and SMA, respectively.

**Fig. 4:**
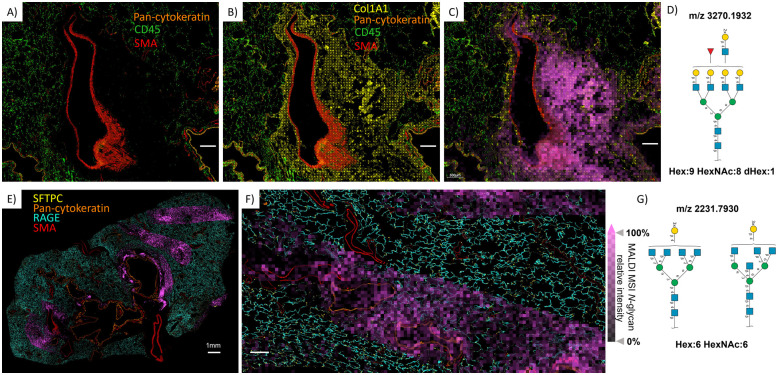
MALDI-MSI *N*-glycans ion images overlaid with CODEX imaging demonstrate region-specific detection of certain *N*-glycans. (A) The CODEX image performed after the spatial *N*-glycomics analysis shows the location of pan-cytokeratin, CD45, and SMA. (B) The same CODEX image as in A with the addition of Col1A1 demonstrates areas of extracellular matrix (ECM) surrounding the pulmonary artery. (C) The localization and abundance of *N*-glycan Hex:9 HexNAc:8 dHex:1 in the collagen-rich ECM regions (magenta pixels). (D) SNFG structure of this complex-fucosylated-polylactosamine-tetra antennary *N*-glycan. (E) and (F) Illustrate Hex:6 HexNAc:6 post-translational modifications in parenchymal alveolar regions, notably not in the region containing *N*-glycan demonstrated in panel C. (G) The SNFG structure of this complex *N*-glycans can be ascribed to as either a tetra antennary or bisecting structure. White scale bars represent 300 μm unless otherwise indicated (panel E).

Inspection of the regions around the pulmonary artery and surrounding parenchyma allowed us to visualize the cellular origin of *N*-glycans with other spatial patterns, Fig. 4. For example, Fig. 4(A)–(C) reveal that MALDI-MS image pixels of complex fucosylated-tetra antennary-polylactosamine *N*-glycans (*m*/*z* 3270.1932) are spread over circular regions containing SMA surrounding the airways and arteries, and hence likely smooth muscle structures, but also over epithelium cells distinguished by high expression of pan-cytokeratin. A Hex:6 HexNAc:6 *N*-glycan that, based on our algorithm, can be ascribed to either as a tetra antennary or bisecting *N*-glycan (Fig. 4(G)), is highly abundant in the alveolar parenchyma (Fig. 4(E) and (F)), which is characterized by high expression of RAGE and SFTPC markers. However, the spatial resolution of our MALDI-MSI does not allow us to exclude the possibility that *N*-glycans with this spatial pattern are also present in the pulmonary surfactant, in the cells of small airways, or in the cells of the blood vessels closely associated with surrounding parenchyma (Fig. 4(E)).

## Discussion

In this study, we highlight the advantages of using CODEX imaging to enhance the biological interpretation of protein *N*-glycosylation across lung tissue with MALDI-MSI. Our innovative technology notably improves the accuracy of attributing specific glycosylation patterns to their cellular origins. Lung tissues are an ideal model for testing this approach due to their intricate composition of closely associated diverse cell types, which often complicates and makes the interpretation of spatial omics data ambiguous. Although our demonstration focuses on single lung tissue, this technology can be readily applied to other human tissues and tissue microarrays (TMA) to address questions related to *N*-glycosylation intercellular heterogeneity, given that both techniques are highly reproducible and have been successfully applied to various tissues.

To maximize the potential of our technology, we also developed an algorithm that classifies *N*-glycans into functional categories based on their compositional data. This enabled us to infer the chemical characteristics of *N*-glycans predominantly associated with proteins in specific functional units of the organ. While our algorithm does not distinguish isomeric structures, it uncovers structural features of protein *N*-glycans that are crucial to their function, biosynthesis, and antigenic properties. One possibility to resolve this ambiguity would be to implement LC-MS/MS after the microdissection of the tissue regions; however, the current limitation in sensitivity (∼20 000 cells) hinders its application at the cellular resolution.^31^

One limitation in MALDI-MS imaging of *N*-glycans is that signal from one pixel (typically, 20 μm × 20 μm to 100 μm × 100 μm) can often contain content of several neighboring cells, either due to the size of laser ablation spot or unavoidable small delocalization of the *N*-glycans during the sample preparation.^23^ While the latest instruments could enable better resolution (down 0.6 μm × 0.6 μm for the transmission mode MALDI-2 source^32^), their application for *N*-glycan imaging was not demonstrated to go beyond 50 μm × 50 μm pixel size.^33^ Moreover, specific pulmonary cells can be extremely thin, such as the alveolar type 1 cells estimated to be 0.1 μm thick to enable the passive diffusion of oxygen.^34,35^ All these factors limit *N*-glycan discovery studies to tissue functional units rather than specific cells, and orthogonal measurements are necessary to retrieve the cellular identity. For example, we previously showed how single-nuclei RNA sequencing from the same patient can help determine the cellular origin of *N*-glycan aberrations observed through MALDI-MSI.^7^ Still, those studies were not performed on the same tissue section, so there was a risk that they would not capture data from the same biochemical states.^7^ On the other hand, our innovative MALDI-MSI-CODEX technology showed for the first time that orthogonal cell information measurements can be performed on the exact same tissue slice and cells where *N*-glycan measurement was performed.

One drawback of our multimodal approach is that it necessitates performing tissue-partially destructive MALDI-MSI before conducting non-destructive CODEX analysis. This process results in visible alterations to the tissue, particularly where the laser ablates the sample, notably in collagenous structures. However, attempts to perform MALDI-MSI after CODEX led to a significant reduction in *N*-glycan MALDI-MSI sensitivity, making post-MALDI CODEX the preferable sequence. It is important to note that correlative *N*-glycan MALDI-MSI and CODEX imaging do not necessarily indicate that the detected *N*-glycans originate from the protein markers, although this possibility is not excluded. Additionally, in some instances, a MALDI-MSI pixel may contain multiple layers of cells identified by CODEX. Thus, further advancements in the spatial resolution of *N*-glycan MALDI-MSI, the sensitivity of spatial glycopeptidomics, and the development of workflows that enable correlation between detected *N*-glycans, their associated proteins, and the enzymes involved in their synthesis are essential to unlock the full potential of this novel multimodal imaging approach.

## Conclusions

The complex architecture and cellular organization of tissues, such as those in the lung, present significant challenges in ascribing the cellular origin of molecules detected in MALDI-MSI data without spatial cell type identification from orthogonal measurements. Traditionally, combining MALDI-MSI with histochemical and limited plex-immunohistochemical assays has been utilized for nearly a decade. Recently, the integration of AmberGen technology, which employs antibodies with photocleavable mass tags, and imaging mass cytometry, which uses metal-conjugated antibodies, with MALDI-MSI, has shown considerable promise.^13^ These highly multiplexed antibody labeling techniques address some challenges, but AmberGen has been hindered by low spatial resolution, while mass cytometry necessitates sophisticated and expensive instruments. This manuscript demonstrates a significant advancement in this approach by enabling MALDI-MSI to be coupled, for the first time, to CODEX, allowing the visualization of 30 or more protein markers *in situ* at cellular resolution, paving the way for more detailed and comprehensive cellular analyses. This breakthrough provides comprehensive insights into the cellular identity captured within each MALDI-MSI pixel. Utilizing this technology, we successfully assigned specific *N*-glycans to distinct cell types within various regions, including the adventitial areas of airways and blood vessels, submucosal glands, cartilaginous structures, smooth muscles, and alveolar parenchyma. Importantly, this technology extends beyond lung tissue, offering potential applications to any human tissue sample where the revelation of *N*-glycan-specific cellular heterogeneity is needed.

## Author contributions

Christopher Anderton, Dusan Velickovic, Geremy Clair, and Gloria S. Pryhuber conceptualized the study. Dusan Velickovic performed MALDI-MSI analysis. Jeffrey Purkerson and Heidie Huyck performed CODEX analysis. Geremy Clair and Harsh Bhotika created an R package and Shiny app for *N*-glycan classification and assisted in data integration. Funding was acquired by Gloria S. Pryhuber, Geremy Clair, Dusan Velickovic, and Christopher Anderton. Dusan Velickovic wrote the first draft of the manuscript. All authors contributed to editing and finalizing the manuscript.

## Conflicts of interest

There are no conflicts to declare.

## Supplementary Material

MO-021-D4MO00230J-s001

## Data Availability

MALDI imaging datasets, together with *N*-glycan annotations, and overlayed CODEX image are available at METASPACE: https://metaspace2020.eu/project/velickovic-2024_MALDI_CODEX. *N*-Glycan MiniOn is available on GitHub (https://github.com/GeremyClair/NglycanMiniOn/). A shiny app for *N*-glycan MiniOn is available on GitHub (https://github.com/GeremyClair/NglycanMiniOn_shiny).
