# Supplementary material for: Integrating N-glycan and CODEX imaging reveal cell-specific protein glycosylation in healthy human lung
Source: Mol Omics. 2025 May 20;21(4):334–42. doi: 10.1039/d4mo00230j (PMC12090982; doi:10.1039/d4mo00230j)
Supplement: MO-021-D4MO00230J-s001 [file MO-021-D4MO00230J-s001.pdf]

Supplementary Information (SI) for Molecular Omics.
This journal is © The Royal Society of Chemistry 2025

Supporting document for

Integrating *N*-glycan and CODEX imaging reveal cell-specific protein glycosylation in healthy human lung

AUTHORS: Dušan Veličković[1], Jeffrey Purkerson[2], Harsh Bhotika[1], Heidie Huyck[2], Geremy Clair[1], Gloria S. Pryhuber[2], Christopher Anderton[1]

AFFILIATIONS: [1] Earth and Biological Sciences Directorate, Pacific Northwest National Laboratory, Richland, Washington. [2] Department of Pediatrics, University of Rochester Medical Center, Rochester, New York

CORRESPONDING AUTHOR: Dušan Veličković, email: dusan.velickovic@pnnl.gov

Table of Contents:

Figure S1. Annotated H&E microscopy image of the serial lung tissue section used in the study.

Figure S2. Representative spatial patterns of *N*-glycans from METASPACE

Figure S3. MALDI-MSI *N*-glycans protocol followed by CODEX highly multiplexed immunofluorescence.

Tables S1. List of N-glycans that belong to each spatial pattern observed in METASPACE

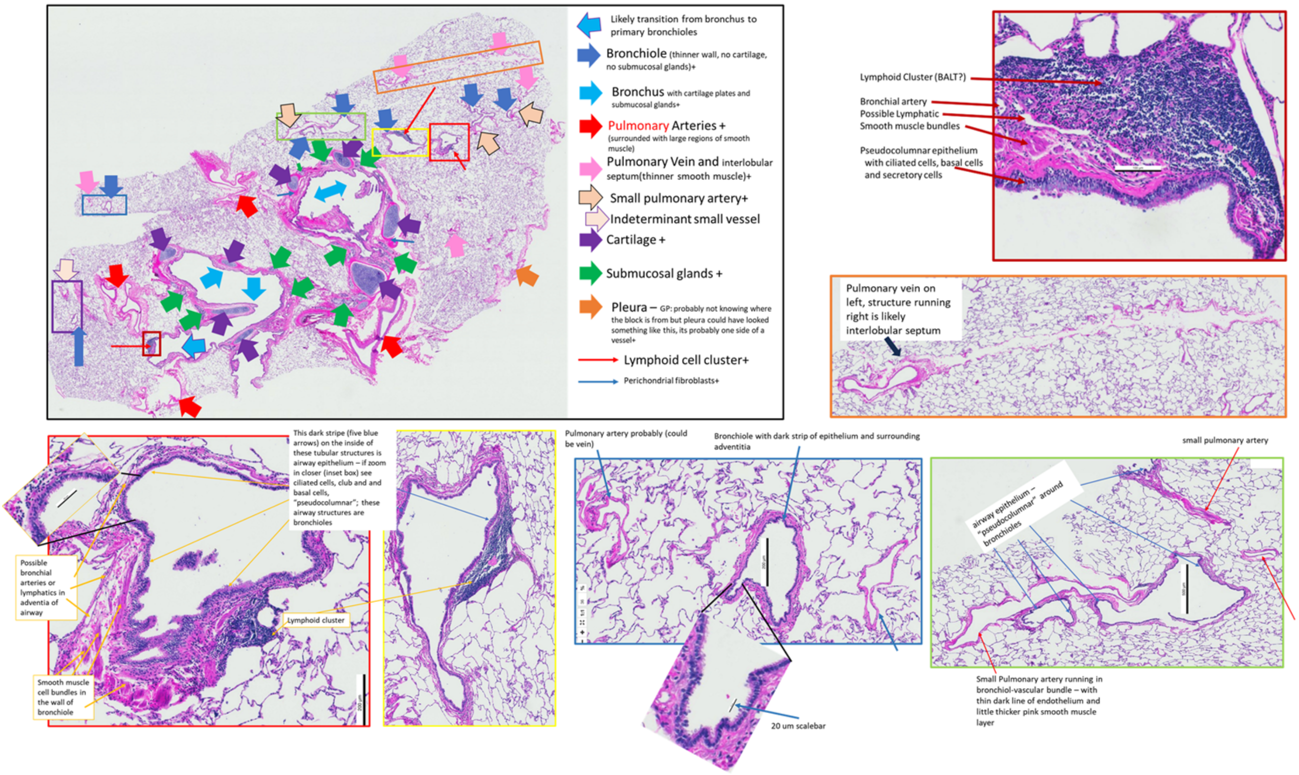

Figure S1.Annotated H&E microscopy image of the serial lung tissue section used in the study. Several regions of interest were zoomed in for better visualization.

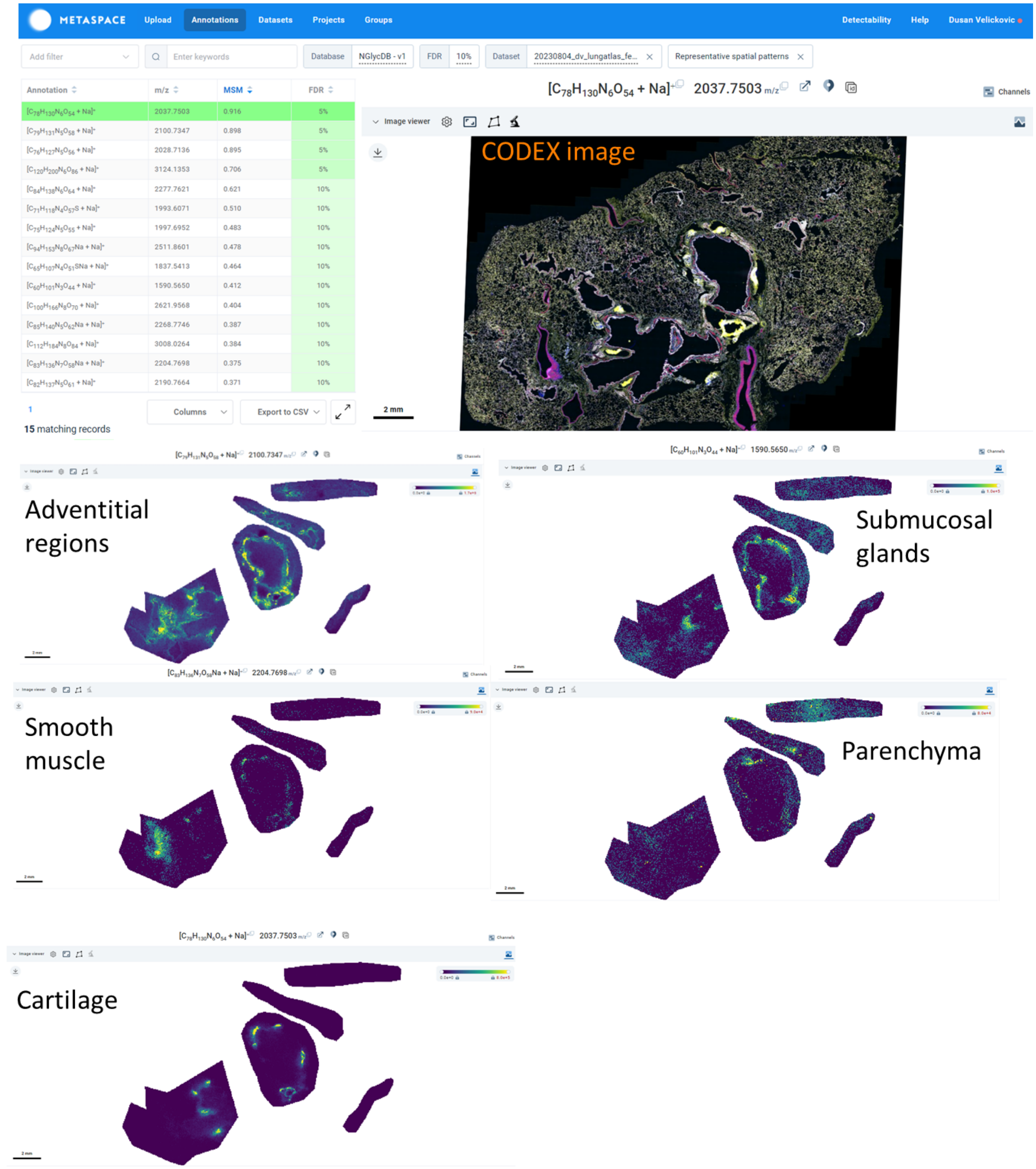

Figure S2. Representative spatial patterns of *N*-glycans from METASPACE. 15 spatial patterns were detected (https://metaspace2020.eu/annotations?db_id=353&ds=2023-08-10_00h34m44s&locs=1), with the five most distinct displayed. A list of glycans that co-localize with those features is presented in **Supporting Table S1**.

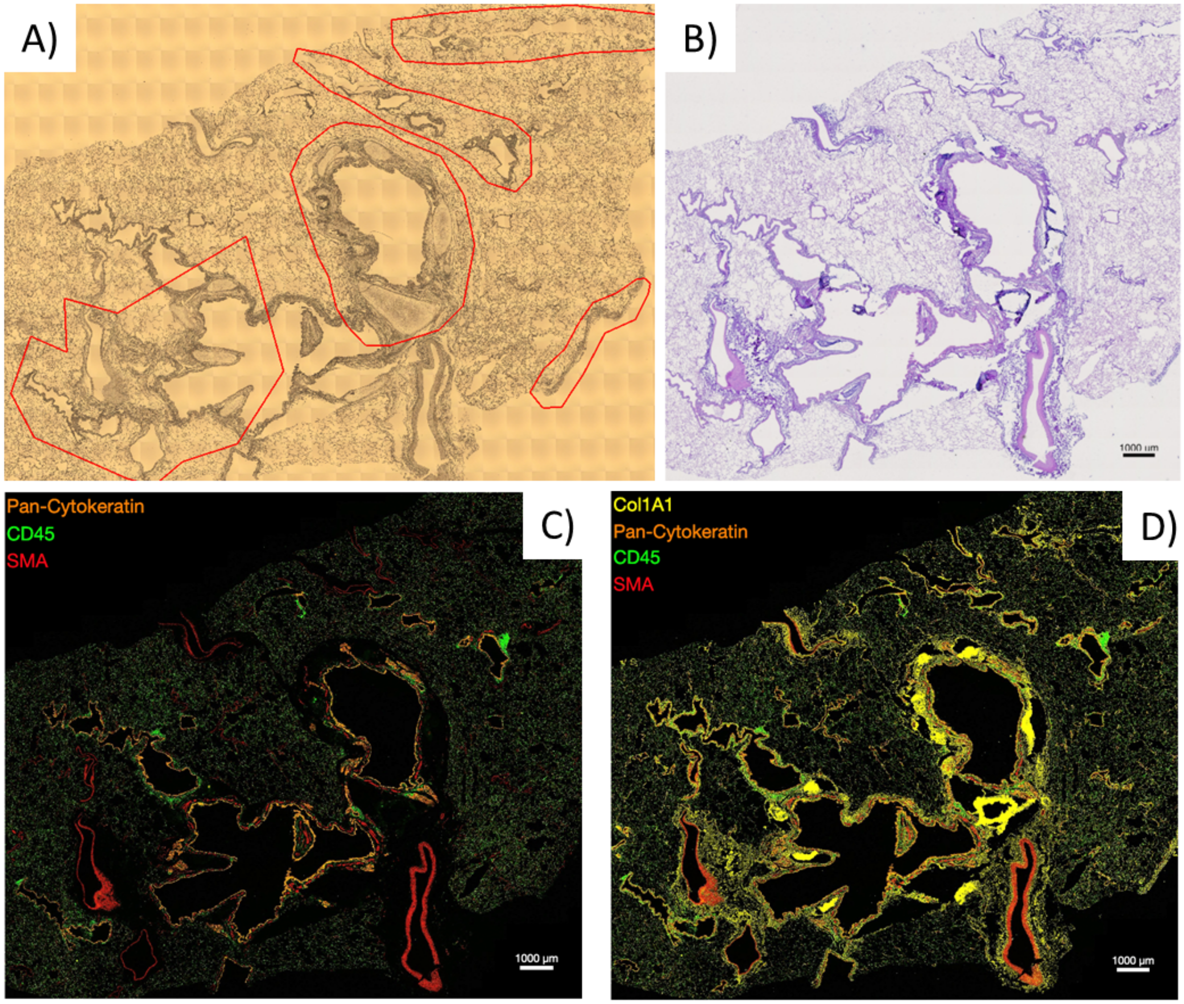

Figure S3. MALDI-MSI *N*-glycans protocol followed by CODEX highly multiplexed immunofluorescence. A) Scout image of lung section with regions of interest to be imaged for N-glycans (red objects) and intact cartilaginous plates. B) H&E performed after N-glycan and CODEX assays demonstrate loss and condensation of cartilaginous plates. C) CODEX image of tissue section following *N*-glycans assay showing antibodies for C) pan-cytokeratin, CD45, and SMA and D) with the addition of Col1A1. Collagen is most notable around broncho-vascular structures and particularly dense in folded cartilaginous plates.

Table S1. List of N-glycans with different spatial patterns observed in METASPACE. All ions were found as [M+Na] or, in the case of some sialic acid N-glycans, as [M-xH+ (x+1)Na] adducts.

**Adventitial regions**		
**m/z**	**N-glycan composition**	**Adduct**
892.2905	Hex:4 HexNAc:1	[M+Na]
917.3221	Hex:2 HexNAc:2 dHex:1	[M+Na]
933.317	Hex:3 HexNAc:2	[M+Na]
974.3435	Hex:2 HexNAc:3	[M+Na]
1079.375	Hex:3 HexNAC:2 dHex:1	[M+Na]
1095.37	Hex:4 HexNAc:2	[M+Na]
1120.402	Hex:2 HexNAc:3 dHex:1	[M+Na]
1136.396	Hex:3 HexNAc:3	[M+Na]
1257.423	Hex:5 HexNAc:2	[M+Na]
1282.454	Hex:3 HexNAc:3 dHex:1	[M+Na]
1298.449	Hex:4 HexNAc:3	[M+Na]
1339.476	Hex:3 HexNAc:4	[M+Na]
1419.476	Hex:6 HexNAc:2	[M+Na]
1444.507	Hex:4 HexNAc:3 dHex:1	[M+Na]
1460.502	Hex:5 HexNAc:3	[M+Na]
1485.534	Hex:3 HexNAc:4 dHex:1	[M+Na]
1501.529	Hex:4 HexNAc:4	[M+Na]
1542.555	Hex:3 HexNAc:5	[M+Na]
1581.528	Hex:7 HexNAc:2	[M+Na]
1605.54	Hex:4 HexNAc:3 NeuGc:1	[M+Na]
1606.56	Hex:5 HexNAc:3 dHex:1	[M+Na]
1622.555	Hex:6 HexNAc:3	[M+Na]
1630.571	Hex:3 HexNAc:4 NeuAc:1	[M+Na]
1647.587	Hex:4 HexNAc:4 dHex:1	[M+Na]
1663.581	Hex:5 HexNAc:4	[M+Na]
1688.613	Hex:3 HexNAc:5 dHex:1	[M+Na]
1704.608	Hex:4 HexNAc:5	[M+Na]
1743.581	Hex:8 HexNAc:2	[M+Na]
1792.624	Hex:4 HexNAc:4 NeuAc:1	[M+Na]
1793.644	Hex:4 HexNAc:4 dHex:2	[M+Na]
1809.639	Hex:5 HexNAc:4 dHex:1	[M+Na]
1850.666	Hex:4 HexNAc:5 dHex:1	[M+Na]
1866.661	Hex:5 HexNAc:5	[M+Na]
1905.634	Hex:9 HexNAc:2	[M+Na]
1938.682	Hex:4 HexNAc:4 dHex:1 NeuAc:1	[M+Na]
1954.677	Hex:5 HexNAc:4 NeuAc:1	[M+Na]

1976.6588		[M-H+2Na]
**Adventitial regions (cont.)**		
**m/z**	**N-glycan composition**	**Adduct**
1955.697	Hex:5 HexNAc:4 dHex:2	[M+Na]
1971.692	Hex:6 HexNAc:4 dHex:1	[M+Na]
1995.703	Hex:4 HexNAc:5 NeuAc:1	[M+Na]
2012.719	Hex:5 HexNAc:5 dHex:1	[M+Na]
2028.714	Hex:6 HexNAc:5	[M+Na]
2067.687	Hex:10 HexNAc:2	[M+Na]
2100.735	Hex:5 HexNac:4 dHex:1 NeuAc:1	[M+Na]
2122.7167		[M-H+2Na]
2101.755	Hex:5 HexNAc:4 dHex:3	[M+Na]
2157.756	Hex:5 HexNAc:5 NeuAc:1	[M+Na]
2158.777	Hex:5 HexNAc:5 dHex:2	[M+Na]
2174.772	Hex:6 HexNAc:5 dHex:1	[M+Na]
2245.772	Hex:5 HexNAc:4 NeuAc:2	[M+Na]
2303.814	Hex:5 HexNAc:5 dHex:1 NeuAc:1	[M+Na]
2325.796		[M-H+2Na]
2304.835	Hex:5 HexNAc:5 dHex:3	[M+Na]
2320.829	Hex:6 HexNAc:5 dHex:2	[M+Na]
2336.824	Hex:7 HexNAc:5 dHex:1	[M+Na]
2341.791	Hex:6 HexNAc:5 NeuAc:1	[M-H+2Na]
2377.851	Hex:6 HexNAc:6 dHex:1	[M+Na]
2391.83	Hex:5 HexNAc:4 dHex:1 NeuAc:2	[M+Na]
2413.8121		[M-H+2Na]
2392.851	Hex:5 HexNAc:4 dHex:3 NeuAc:1	[M+Na]
2414.8325		[M-H+2Na]
2393.846	Hex:7 HexNAc:6	[M+Na]
2466.887	Hex:6 HexNAc:5 dHex:3	[M+Na]
2487.849	Hex:6 HexNAc:5 dHex:1 NeuAc:1	[M-H+2Na]
2522.888	Hex:6 HexNAc:6 NeuAc:1	[M+Na]
2523.909	Hex:6 HexNAc:6 dHex:2	[M+Na]
2537.888	Hex:5 HexNAc:4 dHex:2 NeuAc:2	[M+Na]
2539.904	Hex:7 HexNAc:6 dHex:1	[M+Na]
2668.946	Hex:6 HexNAc:6 dHex:1 NeuAc:1	[M+Na]
2690.928		[M-H+2Na]
2669.967	Hex:6 HexNAc:6 dHex:3	[M+Na]
2756.962	Hex:6 HexNAc:5 dHex:1 NeuAc:2	[M+Na]
2778.9443		[M-H+2Na]
2757.983	Hex:6 HexNAc:5 dHex:3 NeuAc:1	[M+Na]
2779.9647		[M-H+2Na]

2758.978	Hex:8 HexNAc:7	[M+Na]
2816.025	Hex:6 HexNAc:6 dHex:4	[M+Na]
2830.999	Hex:7 HexNAc:6 dHex:1 NeuAc:1	[M+Na]
2852.9811		[M-H+2Na]
2832.02	Hex:7 HexNAc:6 dHex:3	[M+Na]
2887.025	Hex:5 HexNAc:5 dHex:3 NeuAc:2	[M+Na]
2889.041	Hex:7 HexNAc:7 dHex:2	[M+Na]
2903.02	Hex:6 HexNAc:5 dHex:2 NeuAc:2	[M+Na]
2905.036	Hex:8 HexNAc:7 dHex:1	[M+Na]
**Adventitial regions (cont.)**		
**m/z**	**N-glycan composition**	**Adduct**
3035.099	Hex:7 HexNAc:7 dHex:3	[M+Na]
3050.073	Hex:8 HexNAc:7 NeuAc:1	[M+Na]
3122.095	Hex:7 HexNAc:6 dHex:1 NeuAc:2	[M+Na]
3254.173	Hex:8 HexNAc:8 dHex:2	[M+Na]
3269.173	Hex:7 HexNAc:6 dHex:4 NeuAc:1	[M+Na]
**Submucosal glands**		
**m/z**	**N-glycan composition**	**Adduct**
1225.433	Hex:3 HexNAc:2 dHex:2	[M+Na]
1428.512	Hex:3 HexNAc:3 dHex:2	[M+Na]
1589.545	Hex:4 HexNAc:3 NeuAc:1	[M+Na]
1590.565	Hex:4 HexNAc:3 dHex:2	[M+Na]
1768.613	Hex:6 HexNAc:3 dHex:1	[M+Na]
1776.629	Hex:3 HexNAc:4 dHex:1 NeuAc:1	[M+Na]
2246.793	Hex:5 HexNAc:4 dHex:2 NeuAc:1	[M+Na]
2268.7746		[M-H+2Na]
2247.813	Hex:5 HexNAc:4 dHex:4	[M+Na]
2612.945	Hex:6 HexNAc:5 dHex:4	[M+Na]
2978.077	Hex:7 HexNAc:6 dHex:4	[M+Na]
**Cartilage**		
**m/z**	**N-glycan composition**	**Adduct**
1323.4808	Hex:2 HexNAc:4 dHex:1	[M+Na]
1631.5916	Hex:3 HexNAc:4 dHex:2	[M+Na]
1833.6505	Hex:3 HexNAc:5 NeuAc:1	[M+Na]
1745.6345	Hex:3 HexNAc:6	[M+Na]
1891.6924	Hex:3 HexNAc:6 dHex:1	[M+Na]
2037.7503	Hex:3 HexNAc:6 dHex:2	[M+Na]
2183.8082	Hex:3 HexNAc:6 dHex:3	[M+Na]
2297.8511	Hex:3 HexNAc:8 dHex:1	[M+Na]
1996.7238	Hex:4 HexNAc:5 dHex:2	[M+Na]
2053.7452	Hex:4 HexNAc:6 dHex:1	[M+Na]

2110.7667	Hex:4 HexNAc:7	[M+Na]
2256.8246	Hex:4 HexNAc:7 dHex:1	[M+Na]
2069.7401	Hex:5 HexNAc:6	[M+Na]
2215.798	Hex:5 HexNAc:6 dHex:1	[M+Na]
2361.856	Hex:5 HexNAc:6 dHex:2	[M+Na]
2621.9568	Hex:5 HexNAc:8 dHex:1	[M+Na]
2580.9302	Hex:6 HexNAc:7 dHex:1	[M+Na]
3311.1946	Hex:8 HexNAc:9 dHex:1	[M+Na]

**Smooth muscle**		
**m/z**	**N-glycan composition**	**Adduct**
1938.6819	Hex:4 HexNAc:4 dHex:1 NeuAc:1	[M+Na]
1960.6638		[M-H+2Na]
2141.7613	Hex:4 HexNAc:5 dHex:1 NeuAc:1	[M+Na]
2163.7432		[M-H+2Na]
2204.7698	Hex:3 HexNAc:6 dHex:1 NeuAc:1	[M-H+2Na]
3124.1102	Hex:9 HexNAc:8	[M+Na]
3196.1313	Hex:8 HexNAc:7 dHex:1 NeuAc:1	[M+Na]
3218.1132		[M-H+2Na]
3270.1681	Hex:9 HexNAc:8 dHex:1	[M+Na]
3583.2454	Hex:9 HexNAc:8 dHex:1 NeuAc:1	[M-H+2Na]
3635.3003	Hex:10 HexNAc:9 dHex:1	[M+Na]
**Parenchyma**		
**m/z**	**N-glycan composition**	
1825.6342	Hex:6 HexNAc:4	[M+Na]
1987.687	Hex:7 HexNAc:4	[M+Na]
2157.7562	Hex:5 HexNAc:5 NeuAc:1	[M+Na]
2179.7381		[M-H+2Na]
2231.793	Hex:6 HexNAc:6	[M+Na]
2596.9252	Hex:7 HexNAc:7	[M+Na]
2685.9616	Hex:7 HexNAc:6 dHex:2	[M+Na]
2742.9831	Hex:7 HexNAc:7 dHex:1	[M+Na]
3051.0938	Hex:8 HexNAc:7 dHex:2	[M+Na]
3108.1153	Hex:8 HexNAc:8 dHex:1	[M+Na]
